# Serum neutrophil gelatinase-B associated lipocalin (NGAL) levels in Down’s syndrome patients

**DOI:** 10.1186/1742-4933-7-S1-S7

**Published:** 2010-12-16

**Authors:** Giada Dogliotti, Emanuela Galliera, Federico Licastro, Elisa Porcellini, Massimiliano M Corsi

**Affiliations:** 1 Department of Human Morphology and Biomedical Sciences “Città Studi”, Laboratory of Clinical Pathology, University of Milan, Milan, Italy; 2 Department of Experimental Pathology, Laboratory of Immunology, University of Bologna, Bologna, Italy; 3 Laboratory of Biotechnological Applications, IRCCS Istituto Galeazzi, Milan, Italy

## Abstract

Neutrophil gelatinase-associated lipocalin (NGAL) is a group of proteins with different functions.

NGAL is released by different cell types such as epithelial cell, hepatocytes and renal tubular cells during inflammation and after cell injury. Expression of NGAL is induced under various pathophysiological conditions such as infection, cancer, inflammation, kidney injury, cardiovascular disease, burn injury, and intoxication, which has an important anti-apoptotic and anti-inflammatory role.

Subjects with Down’s syndrome (DS) are affected by many pathological age related conditions such as mental retardation, Alzheimer’s disease, immune defects and increased susceptibility to infections. The aim of this study is to evaluate possible use of NGAL as a marker of inflammatory status for allow an early diagnosis of inflammatory disease such as autoimmune disease in DS patients, that are more susceptible to these pathologies, especially in elderly subjects.

In this study were recruited 3 groups of DS subjects (children, adults and elderly) and compared them to healthy control group.

The molecules of interest was determinated by immuno-enzymatic assay (ELISA).

Our results show that NGAL plasmatic level was significantly higher in DS patients compared to healthy controls. Moreover NGAL levels increase in correlation with the age, and showed a significantly correlation between the increase with the severity of disease.

DS is characterized by an enhancement of gene production such as GART, SOD-1 and CBS that encode specific protein and enzyme involved in hydrogen peroxide and superoxide production, species highly cytotoxic implicated in inflammation and ageing.

NGAL may have the potential application to ameliorate the toxicity induced by oxidative stress conditions such as Alzheimer’s disease, thalassemia, cardiovascular disease, burn injury, transplantation, diabetes, and aging.

## Introduction

The neutrophil Gelatinase-associated lipocalin (NGAL) is a member of a large family of lipocalins, a group of small extracellular proteines with great functional diversity [1]. NGAL is found to be constitutively synthesized during a narrow window of maturation in the granulocyte precursors in the bone marrow, and is stored in specific granules of mature neutrophils in complex with gelatinase [2]. NGAL may also be released by epithelial cells, renal tubular cells, and hepatocytes during inflammation or injury, and has been found to be expressed in endothelial cells smooth muscle cells, and macrophages. The aim of this study is to evaluate possible use of NGAL as a marker of inflammatory status for allow an early diagnosis of inflammatory disease such as autoimmune disease in DS patients, that are more susceptible to these pathologies, especially in elderly subjects.

Subjects with DS suffer many medical conditions, among which, cardiovascular disease, the most frequent alteration and the principal cause of mortality; respiratory system pathologies, due to the recurrent inflammatory processes; endocrine hypo-functional alterations; nutritional disturbance; mental retardation and higher risk to develop neuro-degenerative disease, such as Alzheimer’s disease; immune defects and increased susceptibility to infections [3], immune defects of variable degree regarding both specific and non-specific immunity are present in patients with DS and are responsible for the increased incidence of infections (above all lung infections) and autoimmune diseases, in particular of the thyroid and gastro-enteric system.

## Material and methods

Three groups of DS subjects were recruited for this study: the first group consisted of 23 children (age 2-14 years); the second of 14 adults (age 20-50 years) and the third of 13 elderly persons (>60 years) these groups were compared to a group of 20 healthy controls (age 5-60 years). DS children and adults were living at home and the elderly were in institutions. All DS patients were assessed by clinical examination and karyotype analysis; they had mild and variable degrees of mental retardation, no other pathological conditions at the time of the study, and were in good health. The project was approved by the University of Milan Ethics Committee and by the Fondazione Antoniana of Bologna, Italy. The molecule of interest was quantified using immuno-enzymatic assay (R&D System, Minneapolis,USA).

## Results

**Figure 1:**
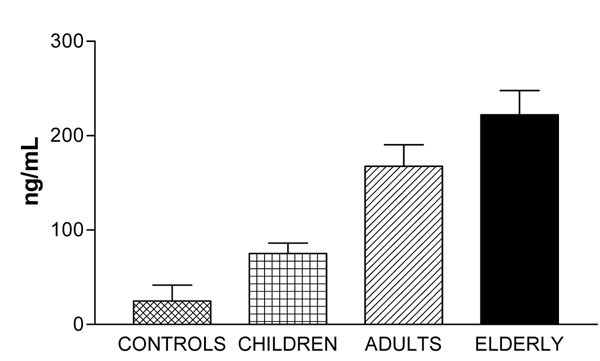
Data show NGAL levels in children, adults and elderly, expressed as mean + SD (ng/mL)

Our data show (Figure 1) an increase of NGAL serum level in Down’s syndrome patients respect to healthy controls. NGAL values in young DS subject show no significantly difference than controls (75.06+48.73 SD ng/mL vs 24.89+33.48 SD ng/mL), while there is a statistical increase in adults (167.51+82.28 SD ng/mL with p<0.01) and elderly ( 222.13+57.73 SD ng/mL with p<0.001) DS subjects than controls. Our data also show that NGAL levels increase in correlation with the age of patients. Middle age subject had significantly higher NGAL values than those from DS children (167.51+82.28 SD ng/mL vs 75.06+48.73 SD ng/mL with p<0.01), differences were also found between elderly subjects and DS children (222.13+57.73 SD ng/mL vs 75.06+48.73 SD ng/mL with p<0.001).

The results showed an increase of the levels of this molecule in our patients respect to a group of healthy controls. Beside we also observed an NGAL age-related increase in Down’s syndrome subjects.

## Discussion

NGAL is thought to mediate inflammatory responses by sequestering neutrophil chemoattractans, particularly N-formylated tripeptides and possibly leukotriene B_4_, and platelet-activating factor.

In literature NGAL has been studied in association with several pathologies, such as, inflammations, autoimmune diseases, cardiovascular and renal injury and also in some cancers, showed a significantly correlation between the increase of NGAL and the course and the severity of the diseases.

Down’s syndrome is characterized by the onset of different clinical conditions, such as mental retardation, endocrine and cardiovascular alterations, immune-defects with possible development of autoimmune diseases, in particular thyroid and gastro-enteric. After the genetic mapping of chromosome 21 has been possible to characterize a long list of genes that encode specific proteins, in particular, genes GART, SOD-1, CBS and the APP which are present in the corresponding region 22 of the long arm of chromosome 21. Genes GART, SOD-1, CBS and APP encode specific proteins and enzymatic activity, respectively involved in the biosynthesis of purine, production of hydrogen peroxide (H_2_O_2_) in the metabolism of sulfur amino acids methionine and cysteine. Increased expression of the GART gene may lead to increased production of superoxide, reactive oxygen species highly cytotoxic.

Oxidative stress has been implicated in several harmful conditions such as Alzheimer’s disease, cardiovascular disease, transplantion, burn injury, inflammation, diabetes and aging [4]. NGAL could act as a cytoprotective factor against H_2_O_2_ toxicity, which is considered to be the classical inducer of oxidative stress caused by ROS generation [5].

For these reasons we wanted to associate NGAL, possible marker of inflammations, with the Down’s syndrome which often presents an inflammatory status. Moreover this study allowed to identify a progressive increase in relation with aging.

In conclusion our pilot study showed that NGAL may have potential application to ameliorate the toxicity induced by oxidative stress and pathological conditions. If present results will be confirmed in a larger sample of DS subjects, NGAL might be used as a marker of inflammation and considered a protective factor against early senescence in subjects with Down’s syndrome.

## Competing interests

The authors declare that they have no competing interests.

## Authors’ contribution

GD and MMC: Conception and design, GD and EP: Interpretation of data, drafting the article EG: Acquisition of data analysis, MMC and FL: Revising it for intellectual content and final approval of the completed article.
